# Identification of a Marine *Bacillus* Strain C5 and Parathion-Methyl Degradation Characteristics of the Extracellular Esterase B1

**DOI:** 10.1155/2014/863094

**Published:** 2014-12-11

**Authors:** Jianhua Hao, Junzhong Liu, Mi Sun

**Affiliations:** Key Laboratory of Sustainable Development of Marine Fisheries, Ministry of Agriculture, Yellow Sea Fisheries Research Institute, Chinese Academy of Fishery Sciences, 106 Nanjing Road, Qingdao, Shandong 266071, China

## Abstract

A bacterial strain C5 that can produce new type of marine esterase was isolated and screened from marine sludge. According to 16S rRNA sequence analysis and physiological and biochemical experiments, the strain was identified as *Bacillus subtilis*. A single isozyme with a molecular weight of 86 kDa was observed by SDS-PAGE and native-PAGE. On this basis, the mechanism of esterase B1 secreted by strain C5 degrading parathion-methyl was explored, and the effects of temperature and pH on the degradation rate were investigated. From the results, *p*-nitrophenol was one of the degradation products of B1 degrading parathion-methyl, and the best degradation effect could be achieved at the temperature of 40°C and the neutral pH value.

## 1. Introduction

In order to adapt to various extreme environments in the ocean, marine microbes can produce corresponding enzymes which have unique physiological functions and enzymology properties [1]. Compared with terrigenous microbial enzymes, the enzymes produced by marine microbes have more potential for further development and application and thus attract more and more attention of the world. Nowadays, developing and looking for enzymes with new properties have become the international hot topics in the study of enzyme preparation [2].

Esterase (EC 3.1) is a kind of hydrolase acting on the ester bond, which can catalyze hydrolysis and synthesis of different substrates. These reactions usually have high substrate specificity and regioselectivity or enantiomorphic selectivity, which make esterase an important biological catalyst in organic synthesis. As a result, esterase has been widely used in food, flavors and fragrances industry, chemical industry, environmental protection fields, and so on [3].

Currently, organic phosphorus is widely used as an ester pesticide, but it is also the main pesticide ingredient that causes poisoning of human beings and animals. There are lots of microbes secreting organophosphorus ester in soil, so that this kind of pesticide is easy to be decomposed in soil [4]. In contrast to the bacteria secreting the enzymes, the organophosphate degradation enzymes are more tolerated to abnormal environmental conditions. In the case of low-concentration pesticide, their degradation effects on the pesticide are far more obvious than the microorganisms themselves since decomposing bacteria can utilize other carbon sources efficiently rather than pesticides [5]. Therefore, degradation enzyme is an effective means of purification and remediation of pesticide-contaminated environment.

According to previous reports, the bacteria that can produce parathion-methyl hydrolase mainly include* Acinetobacter*,* Pseudomonas*,* Burkholderia*,* Arthrobacter*,* Ochrobactrum*, and so on [6–11]. However, the application of* B. subtilis* in pesticide degradation is still very scarce. In this study, a* Bacillus* strain (numbered as C5) that can produce esterase was isolated and screened from marine sludge. Using microbial routine physiological and biochemical detection and molecular biology method, the strain C5 was identified as* Bacillus subtilis*, with GenBank login number as JN942155. Meanwhile, the preliminary degradation mechanism of esterase B1 secreted by strain C5 on parathion-methyl was explored, and the degradation effects were also discussed.

## 2. Materials and Methods

### 2.1. Bacterium Sources and Chemicals

Bacterial strain C5 was isolated from China Bohai Sea (N38°29′42.94′′, E119°23′59.90′′) and stored in Laboratory of Marine Products and Enzyme Engineering, Yellow Sea Fisheries Research Institute, Chinese Academy of Fishery Sciences.

PCR purification kit of UNIQ-10 was purchased from Shanghai Sangon Biological Engineering Technology & Services Co., Ltd. (Shanghai, China), and the related reagents for PCR amplification were from Takara Biotechnology (Dalian) Co., Ltd. (Shanghai, China). 99% parathion-methyl was purchased from Aladdin (Los Angeles, USA). All other reagents were purchased from Sinopharm Chemical Reagent Corp. (Shanghai, China) and were of the highest analytical grade.

#### 2.1.1. Identification of the Extracellular Esterase Producing Bacterial Strain C5

Identification of the strains referred to the common bacterial system identification method. The 16S rDNA sequence of the strain C5 was also identified from the genomic DNA and amplified by PCR using primers 27F (5′-AGAGTTTGATCCTGG TCAG-3′) and 1492R (5′-GGTTACCTTGTTACGA CTT-3′). The amplified product was then purified and sequenced. The nucleotide sequence has been submitted to GenBank under accession number JN942155. The existing sequences of this strain's 16S rRNA sequence were retrieved by BLAST, and the sequence which was genetically close to the testing strain was downloaded. BioEdit software was used to carry on the multiple sequence alignment, and MEGA software was used for phylogenetic analysis by neighbor-joining method.

### 2.2. Purification of Esterase B1

The crude esterase B1 was purified from culture broth using the optimized medium. All purification steps were carried out at 4°C. Esterase B1 in the culture broth was precipitated using a 80% saturated solution of ammonia sulfate. The precipitated protein was dissolved in 0.1 M phosphate buffered solution (pH 7.0) and then dialyzed with the same buffer. Sephadex G-100 (5 gm) gel was added in 0.1 M phosphate buffered solution (pH 7.0) and allowed to swell overnight and column (1.5 × 65 cm) was packed. The column (1.5 × 65 cm) was equilibrated with 0.1 M phosphate buffered solution pH 7.0. Total of 5 mL of ammonia sulfate precipitate was loaded onto a Sephadex G-100 column (1.5 × 65 cm). The column was eluted with phosphate buffered solution (pH 7.0) and fractions of 1 mL were collected at a flow rate of 1 mL/min. The esterase-rich fractions were pooled and stored for subsequent analysis.

### 2.3. The Parathion-Methyl Solution Sample Treatment

The reaction system consisted of 15.6 mL phosphate buffer (pH = 7.0), 0.4 mL parathion-methyl solution (with a concentration of 10 mg/mL, dissolved by methyl alcohol), and 4 mL enzyme solution containing 200 U/mL esterase. This reaction system firstly ran in the shake culture water bath pot for a certain time at 40°C and then suffered a reduced pressure distillation until drying. Afterwards, 20 mL chromatographically pure acetonitrile was added to redissolve the distilment, followed by a membranous examination. The control sample was inactivated enzyme solution.

### 2.4. HPLC and Mass Spectrometry Detection

HPLC detection conditions: the mobile phase was the mixture of methyl alcohol and water with a volume ratio of 80/20; the flow rate was 1.0 mL/min; and the detection wavelength was 274 nm. By an external standard method, pesticide parathion-methyl concentration could be calculated according to the sample peak area, the reference sample peak area, and the sample size.

Mass spectrometry chromatographic conditions: temperature of injection port was 250°C, and detector temperature was 280°C; carrier gas was high purity helium gas, with a flow rate of 1.0 mL/min; column temperature was raised from 50°C to 260°C by program with a rate of 10°C/min and then kept for 3 min. TOF mass spectrometry conditions: quality scan range was 50–500, and full scan mode was used for the detection.

### 2.5. Effect of Temperature and pH on the Degradation Rate

According to the treatment method mentioned above, the degradation effect was detected at the temperatures of 20°C, 30°C, 40°C, and 50°C, respectively. The degradation effect of the enzyme solution on the parathion-methyl was detected in the pH range of 5–8 at 40°C.

### 2.6. Esterase Assay

Esterase activity was assayed by following the release of* p*-nitrophenol (*p*NP) from* p*-nitrophenyl phosphate (*p*NPP). The standard assay was carried out in 1 mL reaction mixture containing 0.04 M PBS buffer (pH 7), 3 mM substrate* p*NPP, and enzyme. After incubation at 30°C for 15 min, the reaction was terminated by adding equal volume of 1 M NaOH, and the released* p*NP in the reaction mixture was measured at 400 nm using a spectrophotometer. One unit of enzyme activity represents the hydrolysis of 1 umol* p*NP from* p*NPP per min under the standard assay.

## 3. Results

### 3.1. Identification of the Extracellular Esterase Producing Bacterial Strain C5

**Figure 1:**
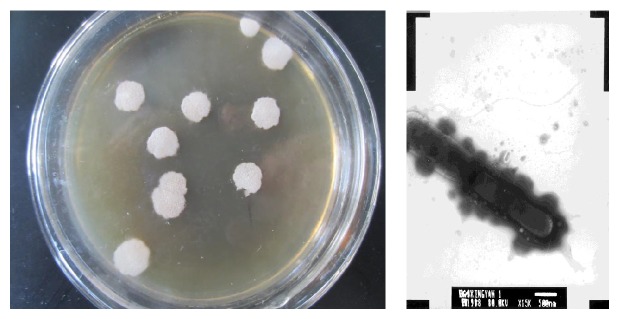
Single colony of strain C5 observed by electron microscope.

Colony of strain C5 on the beef extract peptone medium was white, large, and flat, with rough surface and irregular edges, as shown in Figure 1. The bacterial strain was gram positive and rod shaped, single or arranged as short chain. The size of its thalli was 0.45~0.7 *μ*m × 2.0~3.8 *μ*m, and the spores were centrally or partially accrete, which enlarged the thalli. Electron microscope observation showed that its flagella grew by means of adnation.

Physiological and biochemical identification of the strain C5 were conducted, and the results were shown in Table 1. According to* Bergey's Manual of Systematic Bacteriology* Eighth Edition, all the physiological and biochemical characteristics of* Bacillus* strain C5 were basically the same with* Bacillus subtilis*. Further, combined with the phylogenetic analysis, the strain C5 was identified as* Bacillus subtilis*.

1462 gene bases (JN942155) from 16S rRNA of strain C5 gene were searched for homologous sequences in GenBank. Among the displayed 100 strains with high similarity, 75 belonged to* B. subtilis*, 15 were unidentified, 3 belonged to* B. tequilensis*, 2 belonged to* B. mojavensis*, and the last three belonged to* B. amyloliquefaciens*,* B. licheniformis,* and* B. axarquiensis,* respectively.

**Figure 2:**
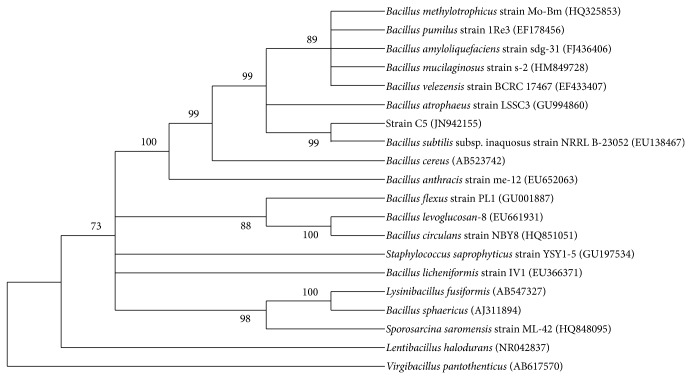
Phylogenetic tree of strain C5 based on the 16S rRNA gene sequence homology (in parentheses were the GenBank login numbers).

Based on the homology of 16S rRNA sequence, strains with different sequence similarity from relative species were selected, and multiple sequence alignment comparison was carried out by BioEdit program. Then, bootstrap values (which were labeled in the figures) were calculated by neighbor-joining analysis method using MEGA software, and phylogenetic tree was constructed as shown in Figure 2. It can be seen that strain C5 had the highest homology up to 99% with* Bacillus subtilis* subsp. inaquosus strain NRRL B-23052. Bacteria taxonomists widely believe that when the homology of 16S rRNA sequence is above 97%, the strains can be considered as the same species and genus, whereas when the homology is lower than 93%–95%, they are likely the members of different genera [12]. Therefore, according to the phylogenetic analysis of strain C5, it could be preliminarily identified as* Bacillus subtilis*.

### 3.2. Purification of Esterase B1

**Figure 3:**
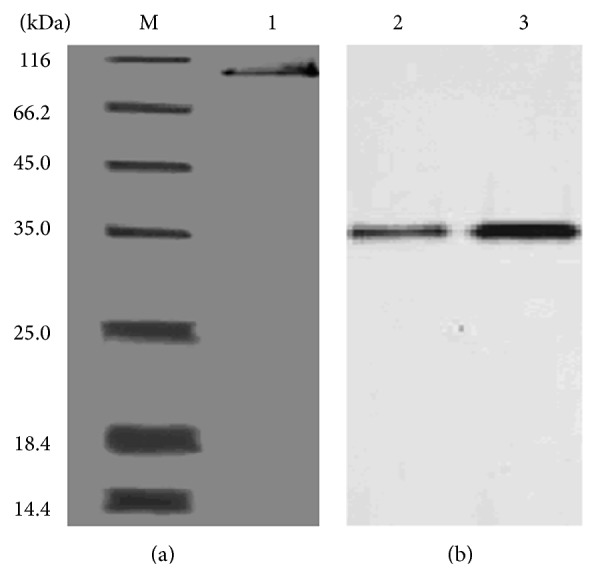
SDS-PAGE and isozyme analysis of esterase purified from C5. (a) SDS-PAGE gel. (b) Native-PAGE gel stained with Fast blue B salt. Lanes: M: molecular mass protein marker, 1: purified esterase, 2: esterase crude extract, and 3: purified esterase.

Purification and gel electrophoresis of esterase B1 that was purified by (NH_4_)_2_SO_4_ precipitation and gel filtration chromatography are summarized in Table 2. The purified esterase B1 was analyzed by SDS-PAGE and native-PAGE, and one major band was observed after PAGE under denaturing and nondenaturing conditions (Figure 3). The results indicated a molecular weight of esterase B1 of 86 kDa.

#### 3.2.1. Preliminary Research on the Mechanism of Parathion-Methyl Degradation by Esterase B1

**Figure 4:**
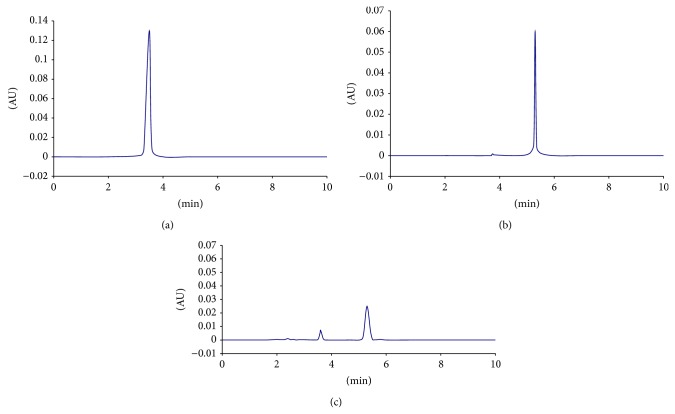
HPLC of parathion-methyl enzymolysis. (a) Standard sample of* p*NP; (b) before enzymolysis of parathion-methyl; (c) after enzymolysis of parathion-methyl.

**Figure 5:**
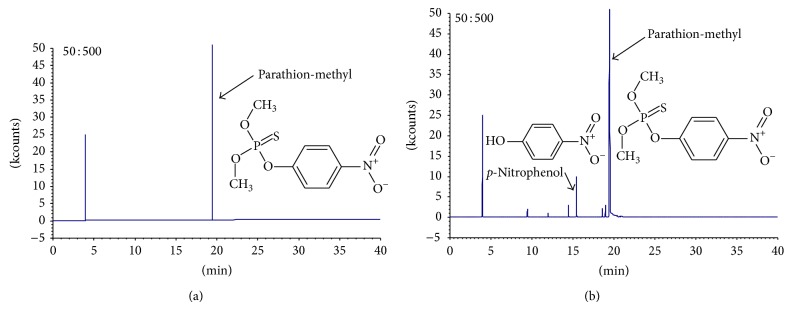
TOF mass spectrogram. (a) Before enzymolysis of parathion-methyl; (b) after enzymolysis of parathion-methyl.

Figure 4 showed the HPLC of parathion-methyl enzymolysis before and after 5 minutes. From the figure, it was obvious that there was* p*NP generated, along with other new products. GC-TOF mass spectrometry was used to further determine the structure of this product, and the results were shown in Figure 5. Undegraded parathion-methyl and generated* p*NP were detected. Besides, there were peaks of other materials that needed further research.

### 3.3. Effect of Temperature and pH on the Degradation Effect

**Figure 6:**
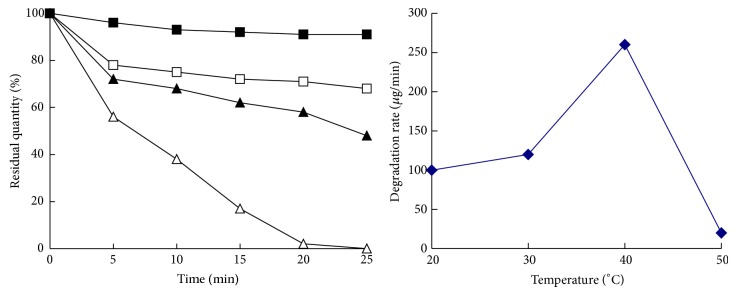
Effect of temperature on the degradation rate. □20°C, ▲30°C, △40°C, and ■50°C.

As shown in Figure 6, the degradation rate of enzyme solution at 40°C was obviously higher than that at other temperatures, and the pesticide parathion-methyl with a concentration of 200 *μ*g/mL could be degraded completely within 20 minutes. However, almost no degradation took place at 50°C, perhaps because the enzyme had been disabled at such high temperature.

**Figure 7:**
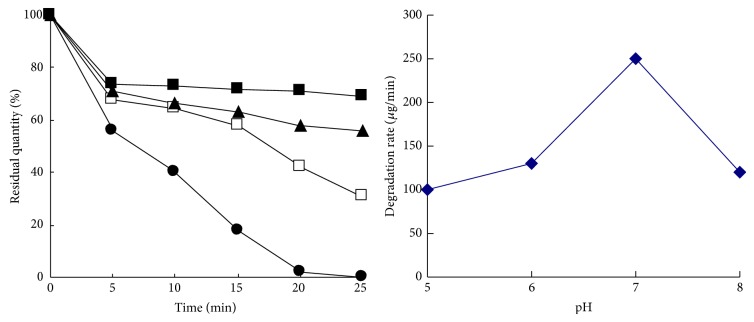
Effect of pH on the degradation rate. ■pH 5, ▲pH6, ●pH7, and □pH8.

As shown in Figure 7, when the pH value of the enzyme solution was 7, the degradation rate was the highest. When the enzyme solution was under acidic or alkaline condition, the hydrolysis of parathion-methyl was accelerated but the degradation rate was reduced. From the results, the pH value had great effects on the degradation rate during the degradation of pesticides, and the degradation rate was the highest under the neutral condition.

### 3.4. Discussions

Study on microbial degradation of pesticides began in the late 40s, and it has always been a hot research spot. The action modes of microbes degrading pesticides mostly belong to enzymatic reactions which are mainly triggered by a microbial intracellular enzyme. For enzymatic reactions, pesticides are firstly adsorbed on the surface of microbial cells, and then the pesticides penetrate cell membranes into the cell interior. Inside the membrane, the pesticides can combine with degrading enzyme and trigger the enzyme catalytic reactions [13–15].

Many researches have been carried out on the mechanism of parathion-methyl biodegradation all over the world. Most reported biodegradations of parathion-methyl were achieved by producing* p*NP and dimethyl thiophosphate (DMTP), but there were also reports of different metabolic pathways. Hou studied the parathion-methyl degradation effect of detoxification enzyme from metabolism of* Pseudomonas alcaligenes* and concluded that parathion-methyl was degraded by detoxification enzyme via P–O bond [16]. Meanwhile, they found that* p*NP and DMTP were produced during the degradation process, but there was no excessive accumulation of* p*NP, indicating that there might be further degradation pathways. Parathion-methyl degradation enzyme screened by Liu et al. could directly mineralize parathion-methyl, and there were no intermediate metabolites or final degradation products [6].* Pseudomonas aeruginosa* screened by Zheng et al. could degrade the parathion-methyl into* p*NP; meanwhile, it could degrade the metabolic intermediate material* p*NP, which avoided the secondary pollution caused by accumulation of intermediate materials [17].* Arthrobacter* sp. isolated by Liu et al. could degrade both parathion-methyl and* p*NP, and almost no* p*NP was detected during the degradation [18]. Hydrolytic enzyme extracted by Liu et al. from parathion-methyl degradation bacteria could degrade the parathion-methyl into* p*NP [6].* Pseudoxanthomonas japonensis* isolated by Feng et al. could degrade both parathion-methyl and* p*NP [19]. DM-1 bacteria isolated by Dong could degrade parathion-methyl into amino parathion-methyl with low toxicity [20]. Esterase B1 secreted by strain C5 could degrade parathion-methyl, and HPLC and GC-TOF mass spectrometry analysis of the products confirmed that* p*NP was one of the degradation products. As for the other product, it might be DMTP, but it still needed further identification.

## 4. Conclusions

In this study, a high activity esterase B1 was obtained by marine bacterium C5. The strain was identified as* Bacillus subtilis*. A single isozyme with a molecular weight of 86 kDa was observed by SDS-PAGE and native-PAGE. The mechanism of esterase B1 degrading parathion-methyl was explored, and the effects of temperature and pH on the degradation rate were investigated.* p*-Nitrophenol was one of the degradation products of B1 degrading parathion-methyl, and the best degradation effect could be achieved at the temperature of 40°C and the neutral pH value.

## Figures and Tables

**Table 1:** Comparison of features between strain C5 and *Bacillus subtilis*.

Characteristics	Bacillus subtilis (ATCC 6051)	C5
Gram stain	+	+
Flagella	Adnation	Adnation
Anaerobism	−	−
Hydrolysis of casein	+	+
Acid from glucose	+	−
Produces gas from glucose	−	−
Voges and Proskauer test	+	−
Methyl red test	−	−
Indole	−	−
Utilization of citrate	+	+
Hydrogen sulfide production	−	−
Hydrolysis of starch	+	−

+: >85% positive; −: 0–15% positive.

**Table 2:** Summary of esterase B1 purification from *Bacillus subtilis* C5.

Purification step	Total activity (U)	Total protein (ug)	Specific activity (U/mg)	Yield (%)	Purification fold
Culture supernatant	75.5	2216.2	34.07	100	1.0
(NH_4_)_2_SO_4_ precipitation	212	1160.85	182.62	56.16	5.36
Sephadex G-100	35.5	63.75	556.86	9.4	16.34

## References

[1] Loperena L., Soria V., Varela H., Lupo S., Bergalli A., Guigou M., Pellegrino A., Bernardo A., Calviño A., Rivas F., Batista S. (2012). Extracellular enzymes produced by microorganisms isolated from maritime Antarctica. *World Journal of Microbiology and Biotechnology*.

[2] Zhang C., Kim S.-K. (2012). Application of marine microbial enzymes in the food and pharmaceutical industries. *Advances in Food and Nutrition Research*.

[3] Asoodeh A., Ghanbari T. (2013). Characterization of an extracellular thermophilic alkaline esterase produced by *Bacillus subtilis*DR8806. *Journal of Molecular Catalysis B: Enzymatic*.

[4] Mandal K., Singh B., Jariyal M., Gupta V. K. (2013). Microbial degradation of fipronil by Bacillus thuringiensis. *Ecotoxicology and Environmental Safety*.

[5] Lu G.-N., Tao X.-Q., Dang Z., Yi X.-Y. (2005). Advances of study on pesticide-degrading strains and their gene engineering. *Bulletin of Mineralogy Petrology and Geochemistry*.

[6] Liu F.-Y., Hong M.-Z., Liu D.-M., Li Y.-W., Shou P.-S., Yan H., Shi G.-Q. (2007). Biodegradation of methyl parathion by Acinetobacter radioresistens USTB-04. *Journal of Environmental Sciences*.

[7] Wang B., Xiong L., Zheng Y., Zhang Z., Tong W., Liu S., Chen Y., Xiao W., Liu D. (2008). Cloning and expression of the mpd gene from a newly isolated methylparathion-degrading strain of bacteria. *Acta Scientiae Circumstantiae*.

[8] Wang X.-X., Chi Z., Ru S.-G., Chi Z.-M. (2012). Genetic surface-display of methyl parathion hydrolase on *Yarrowia lipolytica* for removal of methyl parathion in water. *Biodegradation*.

[9] Ekkhunnatham A., Jongsareejit B., Yamkunthong W., Wichitwechkarn J. (2012). Purification and characterization of methyl parathion hydrolase from Burkholderia cepacia capable of degrading organophosphate insecticides. *World Journal of Microbiology and Biotechnology*.

[10] Kim K.-D., Ahn J.-H., Kim T., Park S. C., Seong C. N., Song H.-G., Ka J.-O. (2009). Genetic and phenotypic diversity of fenitrothion-degrading bacteria isolated from soils. *Journal of Microbiology and Biotechnology*.

[11] Tian J., Wang P., Gao S., Chu X., Wu N., Fan Y. (2010). Enhanced thermostability of methyl parathion hydrolase from *Ochrobactrum* sp. M231 by rational engineering of a glycine to proline mutation. *The FEBS Journal*.

[12] Vandamme P., Pot B., Gillis M., de Vos P., Kersters K., Swings J. (1996). Polyphasic taxonomy, a consensus approach to bacterial systematics. *Microbiological Reviews*.

[13] Boyle A. W., Silvin C. J., Hassett J. P., Nakas J. P., Tanenbaum S. W. (1992). Bacterial PCB biodegradation. *Biodegradation*.

[14] Sato K. (1994). Effect of nutrients on interaction between pesticide penta-chlorophenol and microorganisms in soil bioremediation through rhizosphere technology. *Bioremediation through Rhizosphere Technology*.

[15] Gajbhiye V. T., Agnihotri N. P., Jain H. K. (1989). Cypermethrin residues on tea. *Pesticide Research Journal*.

[16] Hou Y. X., Zhang J. N., Han L. (2011). Degradation analysis of methyl parathion by detoxifying enzyme. *Journal of Agricultural Science and Technology*.

[17] Zheng Y. L., Liu D. L., Chen S. L. (2006). Study on isolation and characteristic identification of a novel high-efficiency degrading strain of methyl parathion. *Research of Environmental Sciences*.

[18] Liu P.-P., Yan Y.-C., Xie X.-P. (2006). The mechanism of purification and degradation of methylparathion-degrading bacterium YL8. *China Environmental Science*.

[19] Feng Z. Z., Liang B., Zhang J. (2010). Degradation characteristics of methyl-parathion by *Pseudoxanthomonas* sp. XY3. *Jiangsu Agricultural Sciences*.

[20] Dong M., Yuan Y. L., Guo X. M. Isolation and identification of methyl parathion degrading strain *Pseudomonas spinosa* and the characteristics of the degradation.

